# Supplementary material for: Hot spot prediction in protein-protein interactions by an ensemble system
Source: BMC Syst Biol. 2018 Dec 31;12(Suppl 9):132. doi: 10.1186/s12918-018-0665-8 (PMC6311905; doi:10.1186/s12918-018-0665-8)
Supplement: Supplementary file 2 — All relevant MATLAB code. A simple Matlab implement of our predictor. (ZIP 19,742 kb) [file 12918_2018_665_MOESM2_ESM.zip › Additional file 2/Read ASA/ASA website/dbMPIKT/4YEBA.html]

 NetSurfP - Protein Surface Accessibility and Secondary Structure Predictions

  

|  |  |  |
| --- | --- | --- |
|  |  | NetSurfP - Protein Surface Accessibility and Secondary Structure Predictions  Technical University of Denmark |

  


---

```
# For publication of results, please cite:
# A generic method for assignment of reliability scores applied to solvent accessibility predictions.
# Bent Petersen, Thomas Nordahl Petersen, Pernille Andersen, Morten Nielsen and Claus Lundegaard
# BMC Structural Biology 2009, 9:51 doi:10.1186/1472-6807-9-51
#
# Column 1: Class assignment - B for buried or E for Exposed - Threshold: 25% exposure, but not based on RSA
# Column 2: Amino acid
# Column 3: Sequence name
# Column 4: Amino acid number
# Column 5: Relative Surface Accessibility - RSA
# Column 6: Absolute Surface Accessibility
# Column 7: Z-fit score for RSA prediction
# Column 8: Probability for Alpha-Helix
# Column 9: Probability for Beta-strand
# Column 10: Probability for Coil
E A  4YEBA                  1    0.827  91.124  -0.711   0.003   0.003   0.994
E P  4YEBA                  2    0.442  62.777  -1.778   0.016   0.005   0.979
E S  4YEBA                  3    0.621  72.723  -1.540   0.058   0.017   0.925
E T  4YEBA                  4    0.516  71.528  -0.748   0.109   0.005   0.886
E D  4YEBA                  5    0.551  79.471  -1.141   0.181   0.016   0.803
E H  4YEBA                  6    0.504  91.678   0.393   0.115   0.016   0.868
B L  4YEBA                  7    0.120  22.045   0.545   0.257   0.016   0.727
E D  4YEBA                  8    0.574  82.699   1.068   0.257   0.016   0.727
E Y  4YEBA                  9    0.508 108.624  -0.234   0.600   0.003   0.397
E K  4YEBA                 10    0.497 102.130   0.309   0.717   0.014   0.269
E D  4YEBA                 11    0.494  71.243  -0.053   0.802   0.014   0.185
E D  4YEBA                 12    0.579  83.362  -0.029   0.802   0.014   0.185
E D  4YEBA                 13    0.504  72.641  -0.130   0.717   0.014   0.269
E D  4YEBA                 14    0.501  72.151  -0.139   0.622   0.015   0.363
E K  4YEBA                 15    0.570 117.228   0.294   0.522   0.016   0.462
E A  4YEBA                 16    0.621  68.478  -0.079   0.339   0.016   0.645
E A  4YEBA                 17    0.433  47.695  -0.353   0.354   0.048   0.598
E A  4YEBA                 18    0.293  32.322  -0.551   0.184   0.043   0.773
E K  4YEBA                 19    0.576 118.442  -0.155   0.118   0.150   0.732
E V  4YEBA                 20    0.492  75.651  -0.485   0.064   0.216   0.721
B F  4YEBA                 21    0.200  40.160  -1.237   0.064   0.216   0.721
E L  4YEBA                 22    0.355  65.019  -1.372   0.018   0.088   0.893
B C  4YEBA                 23    0.084  11.850  -0.706   0.052   0.084   0.864
E P  4YEBA                 24    0.454  64.479  -0.585   0.052   0.084   0.864
B G  4YEBA                 25    0.307  24.129  -1.837   0.064   0.216   0.721
E L  4YEBA                 26    0.207  37.810   0.747   0.022   0.552   0.426
B L  4YEBA                 27    0.054   9.906   0.367   0.021   0.756   0.223
E K  4YEBA                 28    0.282  57.987   0.087   0.021   0.756   0.223
B G  4YEBA                 29    0.171  13.473  -0.345   0.021   0.756   0.223
B V  4YEBA                 30    0.065  10.052  -0.423   0.022   0.552   0.426
B Y  4YEBA                 31    0.314  67.166  -0.873   0.021   0.279   0.699
E Q  4YEBA                 32    0.500  89.229   0.185   0.018   0.088   0.893
B S  4YEBA                 33    0.188  22.034  -1.113   0.064   0.216   0.721
E E  4YEBA                 34    0.407  71.138   0.451   0.021   0.756   0.223
E H  4YEBA                 35    0.331  60.154   0.546   0.011   0.918   0.071
B L  4YEBA                 36    0.215  39.403   0.676   0.011   0.918   0.071
E F  4YEBA                 37    0.331  66.432   0.002   0.018   0.846   0.136
E E  4YEBA                 38    0.455  79.401  -0.142   0.021   0.756   0.223
B S  4YEBA                 39    0.272  31.832  -0.651   0.021   0.451   0.528
E D  4YEBA                 40    0.591  85.192  -1.281   0.019   0.141   0.840
E H  4YEBA                 41    0.433  78.708  -0.922   0.018   0.088   0.893
E Q  4YEBA                 42    0.459  81.960   0.338   0.066   0.296   0.638
E S  4YEBA                 43    0.321  37.645  -0.015   0.022   0.359   0.619
B G  4YEBA                 44    0.054   4.218  -1.415   0.022   0.552   0.426
B A  4YEBA                 45    0.054   5.929  -0.969   0.023   0.655   0.322
B W  4YEBA                 46    0.059  14.165  -0.499   0.023   0.655   0.322
B C  4YEBA                 47    0.065   9.168  -0.320   0.022   0.552   0.426
B K  4YEBA                 48    0.187  38.445   0.357   0.022   0.359   0.619
B D  4YEBA                 49    0.081  11.600  -1.299   0.019   0.141   0.840
B P  4YEBA                 50    0.172  24.378  -1.389   0.018   0.047   0.935
E L  4YEBA                 51    0.377  69.120  -0.428   0.018   0.019   0.964
E Q  4YEBA                 52    0.493  87.978  -0.648   0.018   0.019   0.964
E A  4YEBA                 53    0.631  69.514  -0.738   0.018   0.019   0.964
E S  4YEBA                 54    0.506  59.315  -1.662   0.003   0.003   0.994
E D  4YEBA                 55    0.764 110.164  -0.742   0.005   0.015   0.979
E K  4YEBA                 56    0.357  73.414   0.207   0.005   0.336   0.660
B I  4YEBA                 57    0.171  31.579   0.996   0.003   0.718   0.279
B Y  4YEBA                 58    0.093  19.895   0.793   0.001   0.900   0.099
B Y  4YEBA                 59    0.067  14.211   0.077   0.001   0.959   0.040
B M  4YEBA                 60    0.051  10.165   0.060   0.001   0.900   0.099
B P  4YEBA                 61    0.107  15.112  -1.079   0.003   0.718   0.279
B W  4YEBA                 62    0.134  32.227  -2.422   0.022   0.359   0.619
B T  4YEBA                 63    0.096  13.343  -1.253   0.022   0.359   0.619
B P  4YEBA                 64    0.141  19.951  -1.722   0.021   0.279   0.699
B Y  4YEBA                 65    0.211  45.048  -1.388   0.020   0.205   0.775
B R  4YEBA                 66    0.244  55.990  -0.772   0.020   0.205   0.775
B T  4YEBA                 67    0.149  20.639  -1.844   0.020   0.205   0.775
B D  4YEBA                 68    0.224  32.235  -0.826   0.064   0.216   0.721
B T  4YEBA                 69    0.084  11.692   0.283   0.087   0.683   0.230
B L  4YEBA                 70    0.034   6.152   0.060   0.085   0.775   0.140
B T  4YEBA                 71    0.168  23.288   0.892   0.085   0.775   0.140
B E  4YEBA                 72    0.167  29.227   0.055   0.085   0.775   0.140
B Y  4YEBA                 73    0.061  13.078   0.514   0.022   0.552   0.426
E S  4YEBA                 74    0.548  64.214   0.814   0.020   0.205   0.775
E S  4YEBA                 75    0.313  36.648  -0.080   0.052   0.084   0.864
B K  4YEBA                 76    0.195  40.070   0.442   0.879   0.010   0.111
E D  4YEBA                 77    0.535  77.166   0.304   0.938   0.007   0.055
E D  4YEBA                 78    0.296  42.682   0.985   0.879   0.010   0.111
B F  4YEBA                 79    0.064  12.885  -0.345   0.858   0.002   0.139
E I  4YEBA                 80    0.323  59.700  -0.085   0.782   0.003   0.216
E A  4YEBA                 81    0.591  65.150  -0.902   0.600   0.003   0.397
E G  4YEBA                 82    0.440  34.659  -1.653   0.176   0.004   0.820
E R  4YEBA                 83    0.501 114.683  -0.475   0.018   0.019   0.964
E P  4YEBA                 84    0.468  66.480  -0.933   0.005   0.045   0.951
B T  4YEBA                 85    0.269  37.283  -0.640   0.004   0.197   0.799
E T  4YEBA                 86    0.471  65.272  -0.491   0.004   0.514   0.481
E T  4YEBA                 87    0.495  68.601  -0.029   0.003   0.718   0.279
B Y  4YEBA                 88    0.136  29.149   0.664   0.002   0.816   0.182
E K  4YEBA                 89    0.423  87.073   0.469   0.004   0.616   0.381
B L  4YEBA                 90    0.049   8.954   0.520   0.004   0.197   0.799
E P  4YEBA                 91    0.351  49.807  -0.818   0.018   0.088   0.893
B H  4YEBA                 92    0.272  49.550  -0.430   0.018   0.088   0.893
E R  4YEBA                 93    0.284  64.990  -0.905   0.018   0.088   0.893
B V  4YEBA                 94    0.111  17.045  -1.732   0.019   0.141   0.840
E D  4YEBA                 95    0.240  34.584  -1.126   0.020   0.205   0.775
B G  4YEBA                 96    0.053   4.147  -2.813   0.019   0.141   0.840
B T  4YEBA                 97    0.060   8.267  -0.948   0.018   0.088   0.893
B G  4YEBA                 98    0.083   6.564  -1.707   0.019   0.141   0.840
B F  4YEBA                 99    0.069  13.929   0.431   0.023   0.655   0.322
B V  4YEBA                100    0.031   4.765   0.208   0.011   0.918   0.071
B V  4YEBA                101    0.031   4.842  -0.020   0.011   0.918   0.071
B Y  4YEBA                102    0.093  19.767   0.419   0.002   0.816   0.182
E D  4YEBA                103    0.270  38.965  -0.560   0.004   0.197   0.799
B G  4YEBA                104    0.134  10.546  -1.472   0.004   0.085   0.910
B A  4YEBA                105    0.067   7.405  -0.890   0.004   0.616   0.381
B L  4YEBA                106    0.042   7.709  -0.186   0.018   0.846   0.136
B F  4YEBA                107    0.065  13.045   0.334   0.018   0.846   0.136
B F  4YEBA                108    0.047   9.353   0.181   0.021   0.756   0.223
B N  4YEBA                109    0.055   8.125   0.575   0.022   0.552   0.426
B K  4YEBA                110    0.194  39.885   0.305   0.021   0.451   0.528
B E  4YEBA                111    0.278  48.602  -0.935   0.020   0.205   0.775
E R  4YEBA                112    0.427  97.783  -1.382   0.004   0.085   0.910
E T  4YEBA                113    0.327  45.383  -0.780   0.005   0.045   0.951
E R  4YEBA                114    0.370  84.753  -1.185   0.018   0.088   0.893
B N  4YEBA                115    0.186  27.274   0.239   0.004   0.420   0.576
B I  4YEBA                116    0.030   5.569  -0.225   0.002   0.816   0.182
B V  4YEBA                117    0.035   5.426  -0.225   0.001   0.959   0.040
B K  4YEBA                118    0.187  38.384   0.148   0.001   0.959   0.040
B F  4YEBA                119    0.064  12.765   0.808   0.001   0.900   0.099
E D  4YEBA                120    0.314  45.219   0.194   0.003   0.718   0.279
B L  4YEBA                121    0.065  11.828   0.297   0.005   0.262   0.733
E R  4YEBA                122    0.413  94.554   0.837   0.004   0.514   0.481
E T  4YEBA                123    0.419  58.143  -0.052   0.022   0.552   0.426
E R  4YEBA                124    0.430  98.493  -0.144   0.021   0.451   0.528
B I  4YEBA                125    0.299  55.278  -0.130   0.021   0.756   0.223
E K  4YEBA                126    0.308  63.397   1.050   0.011   0.918   0.071
E S  4YEBA                127    0.326  38.266  -0.084   0.018   0.846   0.136
E G  4YEBA                128    0.363  28.584  -0.590   0.021   0.756   0.223
E E  4YEBA                129    0.394  68.867  -0.109   0.021   0.756   0.223
B A  4YEBA                130    0.229  25.280   0.043   0.021   0.756   0.223
E I  4YEBA                131    0.309  57.257   0.203   0.021   0.451   0.528
B I  4YEBA                132    0.060  11.082   0.533   0.020   0.205   0.775
E A  4YEBA                133    0.460  50.659  -0.323   0.018   0.088   0.893
E N  4YEBA                134    0.440  64.460  -1.481   0.052   0.084   0.864
B A  4YEBA                135    0.196  21.566  -1.636   0.052   0.084   0.864
B N  4YEBA                136    0.254  37.230  -1.417   0.056   0.142   0.802
B Y  4YEBA                137    0.216  46.095  -0.354   0.056   0.142   0.802
E H  4YEBA                138    0.356  64.665  -1.047   0.052   0.084   0.864
E D  4YEBA                139    0.525  75.667  -1.500   0.018   0.088   0.893
B T  4YEBA                140    0.200  27.698  -0.326   0.019   0.141   0.840
B S  4YEBA                141    0.159  18.635  -0.294   0.020   0.205   0.775
B P  4YEBA                142    0.134  18.958  -0.699   0.021   0.279   0.699
B Y  4YEBA                143    0.118  25.195  -0.657   0.022   0.359   0.619
B R  4YEBA                144    0.252  57.777  -1.596   0.021   0.279   0.699
B W  4YEBA                145    0.234  56.181  -2.058   0.020   0.205   0.775
B G  4YEBA                146    0.234  18.384  -1.062   0.018   0.088   0.893
E G  4YEBA                147    0.266  20.926  -1.926   0.018   0.047   0.935
E K  4YEBA                148    0.281  57.843  -0.401   0.019   0.141   0.840
B S  4YEBA                149    0.099  11.650  -0.470   0.020   0.205   0.775
B D  4YEBA                150    0.169  24.310  -0.119   0.021   0.451   0.528
B I  4YEBA                151    0.026   4.718   0.814   0.022   0.552   0.426
B D  4YEBA                152    0.118  16.946   0.124   0.023   0.655   0.322
B L  4YEBA                153    0.036   6.573   0.302   0.021   0.756   0.223
B A  4YEBA                154    0.068   7.494   0.212   0.023   0.655   0.322
B V  4YEBA                155    0.054   8.346   0.262   0.022   0.552   0.426
B D  4YEBA                156    0.169  24.353   0.561   0.021   0.279   0.699
B E  4YEBA                157    0.242  42.312   0.357   0.018   0.088   0.893
E N  4YEBA                158    0.366  53.568   0.617   0.018   0.088   0.893
B G  4YEBA                159    0.048   3.817   0.301   0.005   0.336   0.660
B L  4YEBA                160    0.043   7.782   0.689   0.003   0.718   0.279
B W  4YEBA                161    0.046  10.967   0.520   0.011   0.918   0.071
B V  4YEBA                162    0.022   3.351   1.038   0.001   0.959   0.040
B I  4YEBA                163    0.025   4.569   1.199   0.001   0.959   0.040
B Y  4YEBA                164    0.075  15.963   0.562   0.001   0.900   0.099
B A  4YEBA                165    0.063   6.910   0.039   0.003   0.718   0.279
B T  4YEBA                166    0.121  16.810   0.581   0.005   0.336   0.660
E E  4YEBA                167    0.463  80.816   0.012   0.005   0.262   0.733
E Q  4YEBA                168    0.571 101.891  -0.382   0.004   0.085   0.910
E N  4YEBA                169    0.608  88.953  -1.747   0.018   0.047   0.935
E N  4YEBA                170    0.543  79.539  -0.180   0.004   0.085   0.910
B G  4YEBA                171    0.247  19.470  -0.210   0.004   0.197   0.799
B K  4YEBA                172    0.195  40.111   0.992   0.003   0.718   0.279
B I  4YEBA                173    0.028   5.106   1.002   0.001   0.900   0.099
B V  4YEBA                174    0.054   8.254   0.650   0.001   0.959   0.040
B I  4YEBA                175    0.025   4.662   0.814   0.001   0.959   0.040
B S  4YEBA                176    0.037   4.278  -0.961   0.001   0.959   0.040
B Q  4YEBA                177    0.175  31.291   0.410   0.002   0.816   0.182
B L  4YEBA                178    0.092  16.772   0.499   0.004   0.514   0.481
E N  4YEBA                179    0.238  34.799   1.165   0.004   0.138   0.858
E P  4YEBA                180    0.351  49.878  -0.092   0.018   0.088   0.893
E Y  4YEBA                181    0.575 122.963  -0.533   0.018   0.088   0.893
E T  4YEBA                182    0.318  44.148   0.248   0.019   0.141   0.840
B L  4YEBA                183    0.074  13.568   0.733   0.022   0.359   0.619
E R  4YEBA                184    0.482 110.424   1.218   0.022   0.552   0.426
B I  4YEBA                185    0.177  32.708   0.616   0.021   0.756   0.223
E E  4YEBA                186    0.343  59.870   0.412   0.011   0.918   0.071
B G  4YEBA                187    0.270  21.233   0.175   0.011   0.918   0.071
B T  4YEBA                188    0.278  38.586  -0.185   0.011   0.918   0.071
B W  4YEBA                189    0.103  24.892   0.303   0.011   0.918   0.071
E D  4YEBA                190    0.486  70.047   0.316   0.018   0.846   0.136
B T  4YEBA                191    0.085  11.789   0.037   0.023   0.655   0.322
E A  4YEBA                192    0.477  52.587  -0.691   0.022   0.359   0.619
B Y  4YEBA                193    0.129  27.610   0.580   0.020   0.205   0.775
E D  4YEBA                194    0.431  62.122  -0.207   0.018   0.088   0.893
E K  4YEBA                195    0.301  61.916   0.227   0.113   0.043   0.844
E R  4YEBA                196    0.562 128.721   0.066   0.184   0.043   0.773
E S  4YEBA                197    0.350  40.997   0.729   0.184   0.043   0.773
B A  4YEBA                198    0.049   5.356   0.423   0.278   0.093   0.628
B S  4YEBA                199    0.071   8.368  -1.203   0.278   0.093   0.628
B N  4YEBA                200    0.142  20.774  -0.154   0.199   0.152   0.649
B A  4YEBA                201    0.027   2.931   0.516   0.252   0.423   0.325
B F  4YEBA                202    0.033   6.563   0.192   0.172   0.690   0.138
B M  4YEBA                203    0.033   6.703   0.173   0.072   0.855   0.073
B I  4YEBA                204    0.029   5.402   0.176   0.072   0.855   0.073
B C  4YEBA                205    0.023   3.159   0.829   0.021   0.756   0.223
B G  4YEBA                206    0.036   2.857  -0.582   0.021   0.756   0.223
B I  4YEBA                207    0.048   8.935  -0.095   0.011   0.918   0.071
B L  4YEBA                208    0.026   4.761   0.958   0.011   0.918   0.071
B Y  4YEBA                209    0.042   9.018   0.678   0.018   0.846   0.136
B V  4YEBA                210    0.016   2.521   1.072   0.018   0.846   0.136
B V  4YEBA                211    0.021   3.228   1.034   0.018   0.846   0.136
B K  4YEBA                212    0.176  36.224   0.345   0.021   0.756   0.223
B S  4YEBA                213    0.076   8.895  -1.012   0.022   0.552   0.426
B V  4YEBA                214    0.155  23.900  -0.262   0.022   0.359   0.619
E Y  4YEBA                215    0.279  59.644  -0.650   0.066   0.296   0.638
E E  4YEBA                216    0.421  73.566  -0.810   0.064   0.216   0.721
E D  4YEBA                217    0.503  72.526  -1.280   0.113   0.087   0.800
E D  4YEBA                218    0.458  65.926  -1.293   0.113   0.043   0.844
E D  4YEBA                219    0.570  82.166  -1.957   0.113   0.043   0.844
E N  4YEBA                220    0.542  79.407  -1.685   0.053   0.043   0.903
E E  4YEBA                221    0.471  82.214  -1.493   0.053   0.043   0.903
E A  4YEBA                222    0.390  42.978  -1.742   0.018   0.047   0.935
E T  4YEBA                223    0.464  64.315  -0.700   0.018   0.088   0.893
E G  4YEBA                224    0.426  33.542  -0.291   0.004   0.138   0.858
B N  4YEBA                225    0.188  27.509  -1.090   0.004   0.420   0.576
E K  4YEBA                226    0.243  50.006   0.686   0.001   0.900   0.099
B I  4YEBA                227    0.023   4.200   0.815   0.001   0.959   0.040
B D  4YEBA                228    0.101  14.525   0.245   0.001   0.959   0.040
B Y  4YEBA                229    0.030   6.368   0.831   0.001   0.959   0.040
B I  4YEBA                230    0.025   4.643   0.867   0.001   0.959   0.040
B Y  4YEBA                231    0.046   9.873   0.740   0.001   0.959   0.040
B N  4YEBA                232    0.102  14.889  -0.032   0.002   0.816   0.182
B T  4YEBA                233    0.078  10.832   0.299   0.005   0.336   0.660
E D  4YEBA                234    0.374  53.937  -0.122   0.004   0.138   0.858
E Q  4YEBA                235    0.506  90.389   0.698   0.018   0.047   0.935
E S  4YEBA                236    0.563  65.995  -1.308   0.018   0.047   0.935
E K  4YEBA                237    0.544 111.880   0.675   0.021   0.279   0.699
E D  4YEBA                238    0.480  69.139  -1.126   0.004   0.514   0.481
E S  4YEBA                239    0.344  40.282  -0.027   0.004   0.616   0.381
E L  4YEBA                240    0.389  71.263   0.187   0.004   0.514   0.481
B V  4YEBA                241    0.058   8.884  -0.250   0.021   0.451   0.528
E D  4YEBA                242    0.359  51.746  -0.595   0.023   0.655   0.322
B V  4YEBA                243    0.086  13.295   0.261   0.021   0.756   0.223
B P  4YEBA                244    0.254  36.057  -0.626   0.021   0.756   0.223
B F  4YEBA                245    0.038   7.707   0.089   0.022   0.552   0.426
E P  4YEBA                246    0.457  64.848   0.931   0.020   0.205   0.775
E N  4YEBA                247    0.326  47.726  -1.197   0.053   0.043   0.903
E S  4YEBA                248    0.436  51.111  -1.153   0.053   0.043   0.903
B Y  4YEBA                249    0.326  69.602  -2.298   0.053   0.043   0.903
E Q  4YEBA                250    0.458  81.745  -0.902   0.052   0.084   0.864
E Y  4YEBA                251    0.362  77.381  -0.705   0.064   0.216   0.721
B I  4YEBA                252    0.200  37.018  -0.237   0.021   0.451   0.528
B A  4YEBA                253    0.116  12.816  -1.126   0.022   0.552   0.426
B A  4YEBA                254    0.158  17.434  -0.951   0.022   0.552   0.426
B V  4YEBA                255    0.060   9.191   0.159   0.022   0.552   0.426
E D  4YEBA                256    0.352  50.694  -0.377   0.022   0.552   0.426
B Y  4YEBA                257    0.115  24.682  -0.161   0.021   0.279   0.699
B N  4YEBA                258    0.260  38.108   0.766   0.018   0.047   0.935
B P  4YEBA                259    0.226  32.041  -0.191   0.053   0.043   0.903
E R  4YEBA                260    0.459 105.225   0.032   0.113   0.043   0.844
E D  4YEBA                261    0.420  60.508   0.488   0.052   0.084   0.864
B N  4YEBA                262    0.253  37.083   0.914   0.064   0.216   0.721
B L  4YEBA                263    0.231  42.296   1.233   0.023   0.655   0.322
B L  4YEBA                264    0.033   6.042   0.745   0.018   0.846   0.136
B Y  4YEBA                265    0.059  12.672   1.214   0.018   0.846   0.136
B V  4YEBA                266    0.029   4.396   0.061   0.018   0.846   0.136
B W  4YEBA                267    0.056  13.540   0.645   0.018   0.846   0.136
B N  4YEBA                268    0.130  19.076  -0.611   0.021   0.451   0.528
E N  4YEBA                269    0.315  46.057  -0.008   0.005   0.045   0.951
B Y  4YEBA                270    0.134  28.743  -1.491   0.004   0.085   0.910
B H  4YEBA                271    0.203  37.017   0.324   0.004   0.616   0.381
B V  4YEBA                272    0.069  10.605   0.237   0.001   0.900   0.099
B V  4YEBA                273    0.053   8.100   0.606   0.001   0.959   0.040
B K  4YEBA                274    0.170  35.051   0.611   0.001   0.959   0.040
B Y  4YEBA                275    0.084  17.951   1.303   0.001   0.959   0.040
E S  4YEBA                276    0.293  34.375   0.996   0.001   0.959   0.040
B L  4YEBA                277    0.048   8.770   0.457   0.001   0.900   0.099
E D  4YEBA                278    0.429  61.891   0.944   0.001   0.900   0.099
B F  4YEBA                279    0.109  21.957  -0.192   0.004   0.514   0.481
E G  4YEBA                280    0.417  32.826  -0.144   0.004   0.197   0.799
E P  4YEBA                281    0.485  68.892   0.076   0.018   0.088   0.893
E L  4YEBA                282    0.355  64.946  -0.258   0.018   0.047   0.935
E D  4YEBA                283    0.572  82.411  -0.076   0.018   0.019   0.964
E S  4YEBA                284    0.377  44.231  -0.095   0.018   0.019   0.964
E R  4YEBA                285    0.602 137.927  -0.338   0.018   0.019   0.964
E S  4YEBA                286    0.697  81.747  -1.150   0.058   0.017   0.925
E G  4YEBA                287    0.369  29.017  -1.663   0.018   0.019   0.964
E P  4YEBA                288    0.601  85.225  -1.211   0.018   0.047   0.935
B V  4YEBA                289    0.252  38.656  -1.437   0.053   0.043   0.903
E H  4YEBA                290    0.470  85.548  -1.174   0.018   0.047   0.935
E H  4YEBA                291    0.467  84.965  -0.702   0.018   0.047   0.935
B G  4YEBA                292    0.229  18.022  -2.469   0.056   0.142   0.802
E Q  4YEBA                293    0.329  58.759  -1.222   0.021   0.279   0.699
B V  4YEBA                294    0.261  40.116  -1.586   0.022   0.552   0.426
B S  4YEBA                295    0.273  31.949  -0.899   0.023   0.655   0.322
E Y  4YEBA                296    0.301  64.409  -1.194   0.023   0.655   0.322
E I  4YEBA                297    0.300  55.500  -0.931   0.022   0.552   0.426
E S  4YEBA                298    0.351  41.172  -1.024   0.020   0.205   0.775
B P  4YEBA                299    0.256  36.298  -0.194   0.056   0.142   0.802
E P  4YEBA                300    0.449  63.713  -0.102   0.118   0.150   0.732
B I  4YEBA                301    0.151  27.843  -1.094   0.118   0.150   0.732
E H  4YEBA                302    0.449  81.746  -0.135   0.216   0.235   0.548
E L  4YEBA                303    0.302  55.296  -0.607   0.199   0.152   0.649
E D  4YEBA                304    0.566  81.489  -1.947   0.191   0.086   0.723
E S  4YEBA                305    0.401  46.950  -1.735   0.113   0.043   0.844
E E  4YEBA                306    0.496  86.686  -0.850   0.113   0.043   0.844
E L  4YEBA                307    0.310  56.761  -0.708   0.053   0.043   0.903
E E  4YEBA                308    0.751 131.130  -0.880   0.053   0.043   0.903
E R  4YEBA                309    0.407  93.134  -0.397   0.018   0.019   0.964
B P  4YEBA                310    0.174  24.705  -1.390   0.058   0.017   0.925
B P  4YEBA                311    0.172  24.421  -0.340   0.109   0.005   0.886
B V  4YEBA                312    0.157  24.069  -1.112   0.600   0.003   0.397
E R  4YEBA                313    0.465 106.531   0.537   0.782   0.003   0.216
B G  4YEBA                314    0.241  18.927  -1.102   0.782   0.003   0.216
B I  4YEBA                315    0.072  13.246  -0.238   0.879   0.010   0.111
B L  4YEBA                316    0.140  25.561   0.401   0.879   0.010   0.111
E E  4YEBA                317    0.491  85.725   1.184   0.879   0.010   0.111
B V  4YEBA                318    0.229  35.259  -0.084   0.831   0.044   0.125
B L  4YEBA                319    0.218  39.952  -0.625   0.751   0.050   0.199
E F  4YEBA                320    0.329  65.930  -0.843   0.455   0.046   0.498
E Q  4YEBA                321    0.835 149.202   0.754   0.016   0.005   0.979

Explain the output.  Go back.
```

---
